# Genetic Variations in the Fibronectin 1 Gene (*FN1*) and Risk of Female Reproductive Cancers—A Preliminary Study

**DOI:** 10.3390/ijms27104302

**Published:** 2026-05-12

**Authors:** Piotr Pawlik, Grażyna Kurzawińska, Marcin Ożarowski, Tomasz M. Karpiński, Anna Bogacz, Piotr J. Olbromski, Aleksandra E. Mrozikiewicz, Maciej Brązert, Wiesław Markwitz, Agnieszka Seremak-Mrozikiewicz

**Affiliations:** 1 Department of Obstetrics and Gynecology, District Public Hospital in Poznan, Juraszów 7/19, 60-479 Poznan, Poland; pitpawlik@gmail.com (P.P.); olbromski.piotr@gmail.com (P.J.O.); 2 Department of Perinatology, Poznan University of Medical Sciences, Polna 33, 60-535 Poznan, Poland; gkurzawinska@ump.edu.pl (G.K.); wmarkwitz@ump.edu.pl (W.M.); asm@data.pl (A.S.-M.); 3 Laboratory of Molecular Biology, Department of Perinatology, Poznan University of Medical Sciences, Polna 33, 60-535 Poznan, Poland; 4 Department of Biotechnology, Institute of Natural Fibres and Medicinal Plants—National Research Institute, Wojska Polskiego 71b, 60-630 Poznan, Poland; 5 Department of Medical Microbiology, Medical Faculty, Poznan University of Medical Sciences, Rokietnicka 10, 60-806 Poznan, Poland; tkarpin@ump.edu.pl; 6 Department of Cancer Immunology, Poznan University of Medical Sciences, Rokietnicka Street 8, 60-806 Poznan, Poland; anna.bogacz@ump.edu.pl; 7 Department of Diagnostics and Treatment of Infertility, Poznan University of Medical Sciences, Polna 33, 60-535 Poznan, Poland; a.mrozikiewicz@ump.edu.pl (A.E.M.); maciejbrazert@ump.edu.pl (M.B.)

**Keywords:** SNV, *FN1* gene, gynecologic cancer

## Abstract

We investigated five single-nucleotide variants (SNVs) of the *FN1* gene in female reproductive organ cancers. The proteins expressed by this gene are essential components of the extracellular matrix (ECM) that constitutes the tumor microenvironment (TME). The study group included 208 women diagnosed with cervical, uterine, and ovarian cancers and 208 age-matched cancer-free controls. Genomic DNA from whole blood was used to analyze five intronic SNVs of the *FN1* gene using PCR/RFLP. The results indicate that two of the studied *FN1* gene variants may increase the risk of gynecological cancers in dominant and log-additive models (*p* = 0.048 and *p* = 0.040 for rs6725958, *p* = 0.033 and *p* = 0.038 for rs1968510, respectively). Comparing individual cancer groups with the controls, differences were observed for the ovarian cancer group (rs1968510 *p* = 0.015 and *p* = 0.016, rs6725958 *p* = 0.070 and *p* = 0.037 in dominant and log-additive models, respectively). None of these associations remained statistically significant after Bonferroni correction for multiple testing. Haplotype analyses revealed that the AGATC haplotype, containing minor alleles for rs35343655 and rs6725958, was more frequent in the entire gynecological cancer group (*p* = 0.0036). For individual cancer types, values of *p* = 0.0071 for ovarian, *p* = 0.0028 for endometrial, and *p* = 0.0269 for cervical cancers were obtained; Our preliminary study suggests that the rs6725958 and rs1968510 *FN1* variants may slightly increase the risk of female reproductive system cancers, particularly ovarian cancer. These findings require further validation in larger, independent cohorts and functional studies.

## 1. Introduction

The most common malignant tumors of the female reproductive system, with a significant impact on morbidity and mortality worldwide, are breast, cervical, ovarian, and endometrial cancers. An estimated 34,630 women in the United States will die from genital system cancers and 42,170 from breast cancers in 2025, corresponding to more than 200 deaths per day [1], The incidence rates for ovarian, endometrial, and cervical cancers in Poland exceed the average levels observed in European countries. In 2021, the incidence rates in Poland and Europe were as follows: 38 vs. 27 per 100,000 for endometrial cancer, 23 vs. 16 per 100,000 for ovarian cancer, and 19 vs. 16 per 100,000 for cervical cancer [2]. Malignancies originate from genetic and epigenetic modifications that impair essential cellular functions, including growth control, apoptosis, and tissue homeostasis [3]. The main threat and cause of death for cancer patients is metastasis. The occurrence of cancer metastasis depends strictly on the migratory and invasive potential of primary tumor cells. The invasive nature of cancer is closely related to epithelial–mesenchymal transformation (EMT). This is a key process influenced by stimuli from the tumor microenvironment, such as the composition of the extracellular matrix (ECM). The ECM prepares cells for EMT and metastasis, making it a key topic for understanding cancer progression [4,5,6]. Integrating proteomic data with genome-wide association studies (GWAS) significantly improves the identification of disease-related genes and pathways compared to using either approach alone. Dysregulation of gene expression and genetic susceptibility loci play a key role in female reproductive tract pathologies and ovarian cancer, as confirmed by literature evidence [7,8]. Heidarzadehpilehrood et al. [8] study integrated three independent mRNA datasets and one miRNA dataset to define a consensus EMT-centered transcriptomic signature in epithelial ovarian cancer. This work found a cohesive group of dysregulated genes and miRNAs that converge on developmental WNT and TGFβ/BMP signaling, the extracellular matrix structure, and EMT [8]. One of the key components of the ECM is fibronectin 1 (FN1). This is high molecular weight, 440 kDa, glycoprotein (composed of two smaller, 230–250 kDa, monomers) present in two forms. Soluble dimeric form in plasma (pFN) is predominantly synthesized by liver hepatocytes and circulates in the blood at a high concentration (approximately 300 μg/mL). Second is cellular FN (cFN), produced by a wide variety of cells (fibroblasts, chondrocytes, myocytes, and synovial cells). cFN is locally secreted in a dimeric or multimeric form at the cell surface and in the extracellular matrix. Both FN forms regulate cell attachment and spreading, although the two types exhibit differences in solubility, binding, size, and proteolytically generated fragments. FN1 participates in a variety of cell adhesion and migratory processes, including as host defense, blood coagulation, wound healing, embryogenesis, and metastasis [9,10,11,12]. Human fibronectin gene (*FN1*, Gene ID: 2335) is located on chromosome 2 (Location: 2q35) and composed of 47 exons. The estimated mRNA length is about 7.9 kilobases (kb) long and has three regions of alternative splicing: the extra domains A (EDA) and B (EDB) and the type III connecting segment (IIICS), which can potentially produce up to 20 different transcript variants. Isoforms generated through alternative splicing enable FN1 to perform a variety of biological roles by interacting with ECM components and numerous integrin receptors [11,13,14].

FN1 has been extensively studied for its involvement in tumor initiation, progression, metastasis, and response to its involvement in tumor initiation, progression, metastasis, and response to chemotherapy. Knockdown of FN1 inhibits tumor growth, while its upregulation promotes cell migration, invasion, and cancer viability [15,16]. Based on data provided by The Genotype-Tissue Expression (GTEx) Project and The Cancer Genome Atlas (TCGA) Program, FN1 mRNA exhibits higher expression in primary tumors than in normal tissues in 17 out of 25 cancer types (68%) for 10 or more normal tissue and primary tumor samples. The highest overexpression occurs in breast cancer, glioblastoma multiforme, head and neck squamous cell carcinoma, pancreatic adenocarcinoma, and thyroid cancer [17]. An integrated multiomics analysis conducted by Zhang et al. [18] highlighted FN1 expression as a key factor influencing prognosis and immune resistance in ovarian cancer. High FN1 expression in tumor cells was associated with poor survival, while down-regulation of FN1 inhibited tumor growth by reducing tumor cell aggregation, invasion, and migration. These findings suggest that tumor cells with high FN1 protein expression contribute to resistance to immunotherapy, making FN1 a potential biomarker and therapeutic target for improving ovarian cancer treatment outcomes [18]. The effects of *FN1* gene single nucleotide variations (SNVs) on human health have not been extensively studied. In the study of Matalliotaki et al. [19], a genetic association between the rs1250248 *FN1* and endometriosis at both the genotypic and allelic level was demonstrated [19]. Among the thirty *FN1* polymorphic variants examined, rs6707530 was associated with tumor shape in colorectal cancer [20].

The purpose of this pilot study was to examine the frequencies of alleles and genotypes of single nucleotide variations (SNVs) in the *FN1* gene and to connect them with the risk of female reproductive malignancies.

## 2. Results

### 2.1. Population Characteristics

The current study included 208 cancers patients and 208 age- and gender-matched cancer-free controls. The mean age for cancer patients was 61.78 ± 13.13 years and for controls was 62.82 ± 12.74 years (*p* = 0.413). Similarly, the data analysis did not show significant relationships among the BMI (29.36 ± 6.45 kg/m^2^ in controls and 29.41 ± 6.19 kg/m^2^ in cases at the time of cancer diagnosis, *p* = 0.925). The research employed the BMI at the time of cancer diagnosis because the patients had a range of gynecological cancers and were in various phases of therapy.

Of the cases, there were 128 (62%) ovarian, 50 (24%) endometrial, and 30 (14%) cervical cancers. The mean age varied among patients with gynecological malignancies (*p* < 0.001). The highest was observed in patients with endometrial cancer (EC) 64.28 ± 14.17 years. Among respondents with ovarian and cervical cancer, the mean age was 62.84 ± 11.99 years (OC) and 53.10 ± 12.96 years (CC). Clinical characteristic data for cancer patients were extracted from medical records and are summarized in Table 1.

### 2.2. Analysis of the Association Between SNVs in Cases and Controls

Allele frequencies for all SNVs in the control group were consistent with Hardy–Weinberg equilibrium (*p* > 0.05). The allelic frequencies for cases and controls are shown in Table 2. The allelic distribution of rs35343655, rs3796123, and rs10202709 were similar in women with gynecologic cancers and the control group. The minor allele of the variants rs6725958 and rs1968510 were more frequent in the study group than in the controls (48.80% vs. 42.07%, OR = 1.31, 95%CI: 1.00–1.73, *p* = 0.051 and 12.74% vs. 8.17% in controls, OR = 1.64, 95%CI: 1.04–2.58, *p* = 0.033). However, after Bonferroni correction, this significant effect was lost.

The association between *FN1* SNVs genotypes in women with cancers and controls was analyzed using the logistic regression method and presented in Table 3. Of the five genetic variants analyzed in this work, none showed a statistically significant association with gynecologic cancers risk after Bonferroni correction (*p* > 0.01). However, the results indicate that the rs6725958 and rs1968510 increase the risk of female reproductive system cancers under dominant (AOR = 1.53, 95%CI: 0.98–2.37, *p* = 0.058, AIC = 580.4 for rs6725958 and AOR = 1.71, 95%CI: 1.04–2.82, *p* = 0.034, AIC = 579.5 for rs1968510) and log-additive models (AOR = 1.34, 95%CI: 1.00–1.80, *p* = 0.046, AIC = 580.0 for rs6725958 and AOR = 1.58, 95%CI: 1.02–2.46, *p* = 0.038, AIC = 579.7 for rs1968510).

### 2.3. Stratified Analysis of FN1 Genotypes and Risk of Gynecological Cancers

Table 4 presents a stratified analysis according to the different types of gynecological cancer, which indicates no statistically significant differences in the frequency of individual *FN1* rs10202709, rs3796123, rs6725958, rs1968510, and rs35343655 genotypes between patients with cervical, endometrial, and ovarian cancer (Table 4).

When comparing individual cancer groups with the controls, differences were observed in group ovarian cancer women. For rs1968510, we found AOR = 1.95, 95%CI: 1.12–3.48, *p* = 0.018 in the dominant model (25.8% of OC and 14.9% of controls had GA+AA genotypes) and AOR = 1.77, 95%CI: 1.10–2.86, *p* = 0.019 in the log-additive model. By analyzing the variant rs6725958 in the OC group, we obtained AOR = 1.66, 95%CI: 0.99–2.79, *p* = 0.051 in the dominant and AOR = 1.44, 95%CI: 1.03–2.00, *p* = 0.030 in the log-additive model. However, after applying the Bonferroni correction for multiple tests (*p* < 0.01), no significant associations were observed for rs1968510 and rs6725958 and ovarian cancer.

### 2.4. Association of FN1 Haplotypes with Gynecologic Cancers Risk

**Figure 1:**
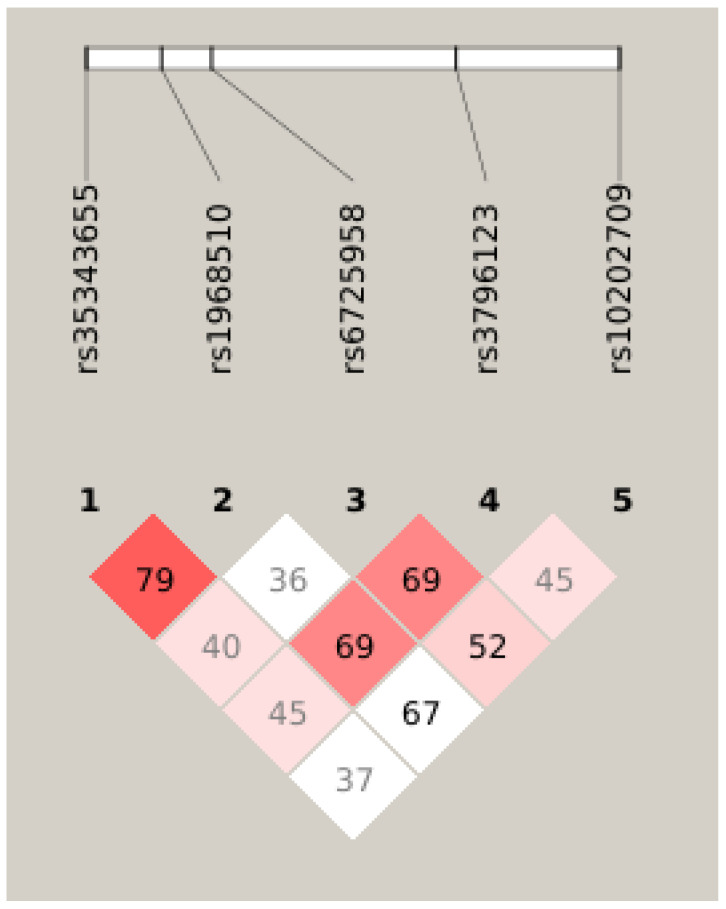
Haploview plot illustrating the linkage disequilibrium of the *FN1* variants in 208 controls and 208 gynecologic cancers in women. The standard linkage disequilibrium color scheme was (D′/LOD) with white to red colors representing the increasing strength of linkage disequilibrium (numbers indicate the D′ value expressed as a percentile).

To measure the degree of linkage disequilibrium between rs35343655, rs1968510, rs6725958, rs3796123, and rs10202709 loci, pairwise LD values (D′ and r^2^) were calculated by the Haploview 4.2 software [19]. Analysis demonstrated limited linkage disequilibrium (LD) among the *FN1* SNVs (Figure 1). One block was constructed spanning 46 kb. The most common haplotype was GGATC (overall frequency 0.310). We then examined whether specific haplotypes of the *FN1* gene increased the risk of developing gynecologic cancers. Interestingly, among the 13 five-marker inferred haplotypes with frequencies = 0.01 in at least one status group, one risk haplotype (AGATC) and one protective haplotype (GGCTC) were identified.

It is worth noting that this protective GGCTC haplotype contains the major alleles of the analyzed polymorphic variants. It occurred statistically significantly more frequently in the control group (10.3% vs. 4.8% in women with gynecological cancers, *p* = 0.0024). The AGATC haplotype, containing minor alleles for rs35343655 and rs6725958, was more frequent in the entire gynecological cancer group (*p* = 0.0036). For individual cancer types, values of *p* = 0.0071 for ovarian, *p* = 0.0028 for endometrial, and *p* = 0.0269 for cervical cancers were obtained (Table 5).

## 3. Discussion

The etiology of gynecological cancers is multifactorial and includes hormonal, environmental, and lifestyle factors. The histologic subtypes within each tumor category of cervical (C53), endometrial (C54), and ovarian cancer (C56) are as follows: (I) carcinoma planoepitheliale cervicis uteri (30); (II) malignant neoplasm of endometrium (5) including adenocarcinoma serosum endometrii (1), adenocarcinoma endometroides endometrii (33), adenocarcinoma clarocellulare (4), carcinoma undifferentiatum endometrii (7), carcinoma solidum undifferentiatum endometrii (5); (III) malignant neoplasm of ovary (128) including adenocarcinoma serosum (33), adenocarcinoma clarocellulare (29), carcinoma solidum (32), cysadenocarcinoma papillare solidum (12), adenocarcinoma mucinosum (22). Several studies also demonstrated the significance of genetic variations in influencing an individual’s chance of contracting the illness. Therefore, this study attempted to find a relationship between five polymorphic variants of the *FN1* gene and the risk of gynecological cancers.

Fibronectin, a key component of the ECM, along with blood vessels, stromal cells, immune cells, signaling substances, and tumor cells all create a complex and dynamic TME. The TME actively shapes tumor initiation, progression, immune evasion, and therapeutic resistance through an intricate network of intercellular communications and molecular signaling [21,22]. The role of FN1 in ECM composition during development, in tissue homeostasis, and cancer is mainly attributable to cFN1. Cellular FN1 consists of a heterogeneous group of isoforms, constituted by a variable proportion of the EDA and EDB domains and of the IIICS, which participate in ECM composition in a tissue-specific manner. It is produced by a variety of cell types, including endothelial cells, chondrocytes, synovial cells, and myocytes, but mainly by fibroblasts [11]. Interestingly, the rs1968510 (NM_212482.4(FN1):c.3797-325T>C) variant in our study suggesting an association with gynecological cancers is located in the intron preceding (−325 bp) exon 25, which encodes EDB. The study of Liu et al. [23] integrated alternative splicing data and gene expression data to identify genes that are regulated by abnormal splicing in glioblastoma multiforme (GBM) progression. The FN1 was the most up-regulated in GBM samples compared with low grade glioma samples and normal brain samples. Used for model construction, FN1 contained four abnormal alternative splicing events (ASEs) resulting in high expression of non-canonical transcripts and the presence of premature termination codon (PTC). An abnormal ASE occurred in exon 25 with additional two bases (AG) retained at the start of the exon, which allowed the PTC in several non-canonical transcripts. Researchers have concluded that PTC may lead to the absence of the EDB domain of exon 25 in truncated proteins and could be involved in GBM acquired immunotherapy resistance [23].

Fibronectin is often studied in connection with various types of cancer, including cancers of the female reproductive tract. Bao et al. [24] demonstrated that the ability of high-grade serous ovarian adenocarcinoma cell line (OVCAR3) to invade and migrate is strongly associated with fibronectin 1 [24]. They propose that FN1 may be a biomarker for ovarian cancer detection and an indicator of its progression. Immunohistochemical analysis of surgical samples of advanced ovarian cancer showed that higher fibronectin expression in the tumor stroma was strongly associated with shorter overall survival (Kaplan–Meier, log-rank test *p* = 0.003) [25]. Yoshida et al. [26] investigated the effect of SPARC (secreted protein acidic and rich in cysteine) expression in endometrial cancer cells on surrounding stromal fibroblasts using an in vitro co-culture system. They observed that SPARC-expressing endometrial cancer cells activated fibroblasts only in the presence of FN1, which was abundantly secreted by the cancer cells. The authors suggest that SPARC- and fibronectin-mediated fibroblast activation may be associated with increased cancer cell motility and invasion [26].

Plasma fibronectin levels have also been examined. Mitev et al. [27] analyzed serum FN levels in women with malignant and benign pathology of the endometrium. The results demonstrated statistical significances (*p* = 0.008) of FN levels in the group with endometrial cancer (mean 482.73, median 409.12 µg/mL) compared to the control group (mean 346.86, median 258.87 µg/mL), but no significant difference in FN levels was observed between the group with endometrial malignancy and the group with benign pathology of the endometrium. In addition, in the cancer group, FN levels did not show any significant differences depending on the histologic type. The authors suggest that serum FN concentration can be used as an additional tumor marker for gynecological malignancies and can be a potential diagnostic and prognostic marker for malignant endometrial pathology as well as for other gynecological malignancies [27]. In the study of Nebioğlu et al. [28], serum FN levels in Turkish patients with non-muscle invasive bladder cancer were determined using the ELISA method. The mean serum fibronectin level in the patient group was 76.794 ± 66.998 ng/mL vs. 50.486 ± 25.156 ng/mL in the control group, and these differences were statistically significant (*p* = 0.003). This study also examined the association of the rs35343655 and rs10202709 variants with plasma fibronectin levels. No significant differences in mean fibronectin levels were found between genotypes. Similarly, no significant differences in response to BCG were found between genotypes for either polymorphism [28].

In our study, the rs1968510 and rs6725958 variants increased the risk of gynecological cancers, particularly ovarian cancer located in intronic regions of the *FN1* gene, which may indicate their potential impact on transcription. The vast majority (nearly 90%) of phenotype-associated SNVs identified from genome-wide association studies (GWAS) are localized in non-coding regions. Some of these variants may be associated only through linkage disequilibrium with the causal SNV, but others may impact transcription factors binding to enhancer elements and alter gene expression. Understanding the functional consequences of genetic variation in the non-coding regions of the human genome remains a challenge [29,30,31]. We performed bioinformatic analyses using RegulomeDB.

This database provides a heuristic ranking (with 1 being the higher and 7 being the lower score) and a probabilistic score (ranging from 0 to 1, with 1 being the most likely to be a regulatory variant) for each query variant representing its computationally predicted regulatory potential. The use of the RegulomeDB database revealed that among the *FN1* gene variants studied in this work, rs1968510 and rs6725958 had the lowest heuristic ranking (5 and 4, respectively), but the highest probabilistic scores (0.590 and 0.609, respectively). Although there is an overall positive correlation between the ranking scores and the model scores, there are some exceptions. These are due to added additional features when predicting model scores and features used in model scoring being weighted differently from ranking scoring [31]. It should be mentioned that prioritizing scores, not functional validation of genetic variants, are provided by the RegulomeDB database that we utilized. Ranking scores 4 and 5 represent TF binding and/or chromatin accessibility peak. However, such scores reflect only limited to moderate evidence of regulatory relevance and do not indicate experimentally validated biological activity.

There is a dearth of research on *FN1* polymorphic variations; we could only find one Finnish study, that examined a variation in the gene’s coding sequence rs2289202 (G>A, exon 3). In a 50-year-old cohort, 810 subjects, of whom 340 had diagnosed hypertension subjects with genotype AA, had significantly more cerebrovascular disease than those with the G allele (*p* < 0.001, OR = 8.73; 95%CI: 2.79–27.31), although those with the A allele had a lower body mass index (*p* = 0.008) [32].

## 4. Materials and Methods

### 4.1. Population

This case-control association study compared a group of women with gynecologic tumors to cancer-free controls. In the study, patients diagnosed with histologically confirmed female reproductive organ cancer according to the International Federation of Gynecology and Obstetrics (FIGO) classification were recruited as cases. According to the ICD-10 classification, the cases were as follows: cervical cancer (C53), uterine cancer (C54), and ovarian cancer (C56). Moreover, healthy age-matched women served as controls. The following exclusion criteria for controls such as smoking, alcohol consumption, or a family history of cancer were used. After complete description of the study to the subjects, written informed consent was obtained. The research was approved by the Local Bioethics Committee at the University Hospital of Medical Sciences in Poznan (No: 1128/18, from 7 November 2018). The study was performed in compliance with the Code of Ethics of the World Medical Association (Declaration of Helsinki). The females, classified in the project, were all hospitalized at the University Hospital of Medical Sciences in Poznan, Poland, between 2019 and 2023.

### 4.2. SNVs Selection and Bioinformatic Analysis

**Figure 2:**
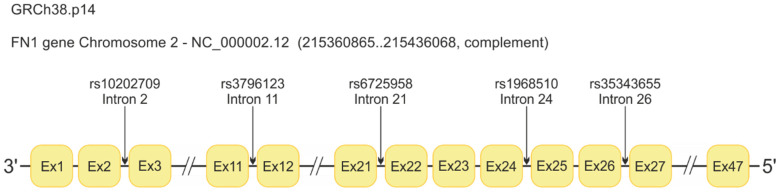
Schematic illustration of the *FN1* gene and the polymorphic variants studied.

Polymorphic variations of the *FN1* gene with a minor allele frequency (MAF) of ≥5% in the Caucasian population were chosen using the NCBI Database of Short Genetic Variations (dbSNP) (https://www.ncbi.nlm.nih.gov/snp, accessed on 1 April 2024) and Database of the 1000 Genomes Project (https://www.ncbi.nlm.nih.gov/variation/tools/1000genomes/, accessed on 17 April 2024). Table 6 includes the dbSNP number, the chromosome position, the alleles of each SNP, and the clinical significance reported in the ClinVar database (https://www.ncbi.nlm.nih.gov/clinvar/variation, accessed on 14 April 2024). The allele frequency was described for European populations in the 1000Genomes Project and the functional rank and score were according to the Regulome Database (https://regulomedb.org/, accessed on 10 June 2024). The location of the variants analyzed in the *FN1* gene is schematically presented in Figure 2.

The functional consequences of intronic *FN1* gene variants were examined in RegulomeDB version 2.2 [31,33]. Because most genetic variants associated with diseases are in non-coding regions of the genome, this database offers a comprehensive platform for analyzing and interpreting these variants. RegulomeDB returns a summary table of variant scores, including a ranking score (where 1 indicates a higher score and 7 indicates a lower score) and a probabilistic score indicating whether the variant may contribute to regulatory variation. Table 6 presents the results of both scoring systems (heuristic and probabilistic). However, to prioritize variants, the heuristic scoring system that we chose was based on the functional confidence of a variant. Category 1 (a–f) indicate that the variant likely affects binding and is associated with target gene expression, whereas ranks 4 to 6 provide no evidence that the variant actually disrupts the binding site [33].

### 4.3. Extraction of DNA and Genotyping

Venous blood samples of women were collected into tubes containing ethylenediaminetetraacetic acid (EDTA) (Sarstedt AG & Co., Nümbrecht, Germany) for DNA extraction. Genomic DNA was obtained using a QIAamp DNA Blood Mini Kit (Qiagen GmbH, Hilden, Germany) according to the guidance from the manufacturer. The quality and quantity of DNA were estimated spectrophotometrically with NanoDrop 2000 (Thermo Scientific, Waltham, MA, USA). Following the standard procedure, samples of DNA were characterized with the A260 nm/A280 nm ratio, which was in the range of 1.8–2.0.

Genotyping of *FN1* rs10202709, rs3796123, rs6725958, rs1968510, and rs35343655 was performed using the PCR-RFLP method [34,35,36]. The sequences of the forward and reverse primers restriction enzymes for each SNVs are summarized in Table 7. Moreover, 10% of subjects selected randomly were tested again to ensure quality control.

### 4.4. Sample Size Calculation

The statistical power using the genpwr R package version 1.0.4 was calculated [37]. Subjects numbering 416 were included in the calculation and the balanced case-control design (1:1 ratio), with a mean MAFs of 0.24. Assuming a logistic model, the significance level was set at 0.05 and a study power of 80% with a detectable odds ratio of 1.85.

### 4.5. Statistical Analysis

Statistical analysis was carried out using the R software version 4.5.2 (R Core Team 2025), https://www.R-project.org/) [38], with the Shapiro–Wilk test assessing data normality. Quantitative data are shown as the mean ± SD, and comparisons between the 2 groups were performed using Student’s *t* test. Qualitative features are shown in the tables in the form of numbers (n) and percentages (%). Two-side *p*-values less than 0.05 were considered statistically significant. Genotype frequency distributions were calculated using five models of inheritance (codominant, dominant, recessive, overdominant, and log-additive) and the SNPassoc package version 2.1-2 [39]. Odds ratios (ORs) and 95% confidence intervals (CIs) were evaluated by logistic regression with adjustment for age and BMI (AOR). The best genetic model was selected based on the Akaike information criterion (AIC). Estimation of haplotype frequency and haplotype association study were conducted using Haploview 4.2 (http://www.broad.mit.edu/mpg/haploview/, accessed on 14 April 2024) software [40]. Furthermore, the statistical significance under multiple testing was corrected by Bonferroni correction. For the 5 selected SNVs, a *p*-value < 0.01 (0.05 divided by 5) was considered statistically significant.

## 5. Conclusions

In conclusion, this research investigated the relationship between genetic variations in the *FN1* gene and the risk of female reproductive cancers. The role of *FN1* in cancer has been found to be inconsistent and not entirely understood. Our results suggest a possible association of rs1968510 and rs6725958 with ovarian cancer. During the statistical analysis, all the ORs and 95% CIs were adjusted by age and BMI in logistic regression analysis to reduce the confounding bias. However, we did not obtain statistical significance after Bonferroni correction. Analysis among the 13 five-marker inferred haplotypes with frequencies = 0.01 in at least one status group, one cancer risk haplotype (AGATC), and one protective (GGCTC) were identified. This study has several limitations that should not be ignored. First, the sample size was restricted, especially in the stratification cancers subgroup and the findings’ generalizability was limited by the data collection from a single center. Second, the present study examined only five SNVs which may not be sufficient to comprehensively evaluate the potential associations of *FN1* polymorphisms with gynecologic cancers. Studies on multiethnic populations and larger samples may clarify the association of *FN1* gene variants with gynecologic cancers. Furthermore, more research is required to determine the biological function of the investigated intronic variations. Prioritization of the rs1 and rs2 variants using the RegulomeDB database revealed no evidence that these variants actually disrupt the binding site and therefore did not demonstrate their experimentally confirmed biological activity. Additionally, it is highly likely that linkage disequilibrium with the causative SNV is the only way in which these variations are linked to ovarian cancer. Finally, this study did not completely account for environmental factors and gene-environment interactions, which may have an impact on the discovered genetic relationships.

## Figures and Tables

**Table 1:** Description of the analyzed ovarian (OC), endometrial (EC), and cervical cancer (CC) in women.

Characteristic	OC (n = 128)	EC (n = 50)	CC (n = 30)	*p* Value
Age (years)	62.84 ± 11.99	64.28 ± 14.17	53.10 ± 12.96	<0.001
Age at diagnosis (years)	60.98 ± 12.06	63.38 ± 14.09	52.00 ± 12.87	<0.001
BMI at diagnosis [kg/m^2^]	28.61 ± 6.47	31.58 ± 5.01	29.23 ± 6.04	0.015
Cancer duration				0.027
≤1 year	82 (64.06%)	43 (86.00%)	24 (80.00%)	
2–4 years	26 (20.31%)	4 (8.00%)	5 (16.67%)	
≥5 years	20 (15.62%)	3 (6.00%)	1 (3.33%)	

**Table 2:** Allelic association of *FN1* gene SNVs.

SNV	Allele	Controls n = 416	Cases n = 416	OR (CI 95%)	*p*-Value (Wald’s Test)	HWE Control *p*-Value
rs35343655	G A	321 (77.16%) 95 (22.84%)	306 (73.56%) 110 (26.44%)	1.21 (0.89–1.67)	0.228	0.694
rs1968510	G A	382 (91.83%) 34 (8.17%)	363 (87.26%) 53 (12.74%)	1.64 (1.04–2.58)	0.033	0.141
rs6725958	C A	241 (57.93%) 175 (42.07%)	213 (51.20%) 203 (48.80%)	1.31 (1.00–1.73)	0.051	0.321
rs3796123	T A	312 (75.00%) 104 (25.00%)	301 (72.36%) 115 (27.64%)	1.15 (0.84,1.56)	0.387	0.355
rs10202709	C T	318 (76.44%) 98 (23.56%)	328 (78.85%) 88 (21.15%)	0.87 (0.63–1.21)	0.406	0.180

HWE—Hardy–Weinberg Equilibrium, OR, odds ratio; CI: confidence interval.

**Table 3:** Distribution of *FN1* genotypes in gynecological cancer patients and controls.

SNV	Model	Genotypes	Controls (N = 208)	Cases (N = 208)	OR (95%CI)	*p*-Value	AIC	AOR (95%CI)	*p*-Value	AIC
rs35343655	Codominant	GG	125 (60.1)	112 (53.8)	1.00	0.436	581.0	1.00	0.464	584.5
		GA	71 (34.1)	82 (39.4)	1.29 (0.86–1.94)			1.27 (0.84–1.92)		
		AA	12 (5.8)	14 (6.7)	1.30 (0.58–2.93)			1.32 (0.58–2.98)		
	Dominant	GA+AA	83 (39.9)	96 (46.2)	1.29 (0.87–1.9)	0.198	579.0	1.28 (0.87–1.89)	0.216	582.5
	Recessive	GG+GA	196 (94.2)	194 (93.3)	1.18 (0.53–2.61)	0.685	580.5	1.21 (0.54–2.68)	0.646	583.8
	Overdominant	GG+AA	137 (65.9)	126 (60.6)	1.26 (0.84–1.87)	0.263	579.4	1.24 (0.83–1.85)	0.297	582.9
	log-Additive	0,1,2	208 (50.0)	208 (50.0)	1.21 (0.88–1.66)	0.229	579.3	1.21 (0.88–1.66)	0.239	582.6
rs1968510	Codominant	GG	177 (85.1)	160 (76.9)	1.00	0.102	578.1	1.00	0.104	581.5
		GA	28 (13.5)	43 (20.7)	1.70 (1.01–2.86)			1.69 (1.00–2.85)		
		AA	3 (1.4)	5 (2.4)	1.84 (0.43–7.84)			1.88 (0.44–8.03)		
	Dominant	GA+AA	31 (14.9)	48 (23.1)	1.71 (1.04–2.82)	0.033	576.2	1.71 (1.04–2.82)	0.034	579.5
	Recessive	GG+GA	205 (98.6)	203 (97.6)	1.68 (0.40–7.13)	0.473	580.2	1.72 (0.41–7.32)	0.454	583.5
	Overdominant	GG+AA	180 (86.5)	165 (79.3)	1.68 (1.00–2.82)	0.050	576.9	1.67 (0.99–2.81)	0.052	580.3
	log-Additive	0,1,2	208 (50.0)	208 (50.0)	1.58 (1.02–2.45)	0.038	576.4	1.58 (1.02–2.46)	0.038	579.7
rs6725958	Codominant	CC	66 (31.7)	48 (23.1)	1.00	0.108	578.2	1.00	0.125	581.9
		CA	109 (52.4)	117 (56.2)	1.48 (0.94–2.32)			1.45 (0.92–2.29)		
		AA	33 (15.9)	43 (20.7)	1.79 (1.00–3.22)			1.77 (0.99–3.19)		
	Dominant	CA+AA	142 (68.3)	160 (76.9)	1.55 (1.00–2.39)	0.048	576.8	1.53 (0.98–2.37)	0.058	580.4
	Recessive	CC+CA	175 (84.1)	165 (79.3)	1.38 (0.84–2.28)	0.204	579.1	1.39 (0.84–2.29)	0.201	582.4
	Overdominant	CC+AA	99 (47.6)	91 (43.8)	1.17 (0.79–1.72)	0.431	580.1	1.15 (0.78–1.69)	0.488	583.5
	log-Additive	0,1,2	208 (50.0)	208 (50.0)	1.35 (1.01–1.81)	0.040	576.5	1.34 (1.00–1.80)	0.046	580.0
rs3796123	Codominant	TT	114 (54.8)	101 (48.6)	1.00	0.326	580.5	1.00	0.356	583.9
		TA	84 (40.4)	99 (47.6)	1.33 (0.90–1.98)			1.31 (0.88–1.96)		
		AA	10 (4.8)	8 (3.8)	0.90 (0.34–2.38)			0.90 (0.34–2.36)		
	Dominant	TA+AA	94 (45.2)	107 (51.4)	1.28 (0.87–1.89)	0.202	579.1	1.27 (0.86–1.87)	0.225	582.5
	Recessive	TT+TA	198 (95.2)	200 (96.2)	0.79 (0.31–2.05)	0.630	580.5	0.79 (0.30–2.05)	0.624	583.8
	Overdominant	TT+AA	124 (59.6)	109 (52.4)	1.34 (0.91–1.98)	0.138	578.5	1.33 (0.90–1.96)	0.155	582.0
	log-Additive	0,1,2	208 (50.0)	208 (50.0)	1.17 (0.84–1.63)	0.352	579.8	1.16 (0.83–1.62)	0.382	583.3
rs10202709	Codominant	CC	125 (60.1)	131 (63)	1.00	0.674	581.9	1.00	0.736	585.4
		CT	68 (32.7)	66 (31.7)	0.93 (0.61–1.41)			0.93 (0.61–1.42)		
		TT	15 (7.2)	11 (5.3)	0.70 (0.31–1.58)			0.73 (0.32–1.66)		
	Dominant	CT+TT	83 (39.9)	77 (37)	0.89 (0.6–1.31)	0.545	580.3	0.90 (0.60–1.33)	0.590	583.7
	Recessive	CC+CT	193 (92.8)	197 (94.7)	0.72 (0.32–1.6)	0.417	580.0	0.75 (0.33–1.68)	0.478	583.5
	Overdominant	CC+TT	140 (67.3)	142 (68.3)	0.96 (0.63–1.44)	0.834	580.7	0.96 (0.64–1.45)	0.845	584.0
	log-Additive	0,1,2	208 (50.0)	208 (50.0)	0.88 (0.64–1.20)	0.421	580.1	0.89 (0.65–1.22)	0.476	583.5

OR, odds ratio; CI: confidence interval; *p*-values were calculated using logistic regression—less than 0.01 was taken as a Bonferroni-corrected significance level, AOR, age and BMI adjusted odds ratio.

**Table 4:** Distribution of *FN1* genotypes in groups of women with cervical, endometrial, and ovarian cancer.

SNV	Genotypes	CC (N = 30)	EC (N = 50)	OC (N = 128)	*p*-Value
rs35343655	GG	17 (56.67%)	24 (48.00%)	71 (55.47%)	0.753
	GA	12 (40.00%)	21 (42.00%)	49 (38.28%)	
	AA	1 (3.33%)	5 (10.00%)	8 (6.25%)	
rs1968510	GG	24 (80.00%)	41 (82.00%)	95 (74.22%)	0.711
	GA	6 (20.00%)	8 (16.00%)	29 (22.66%)	
	AA	0 (0.0%)	1 (2.0%)	4 (3.12%)	
rs6725958	CC	7 (23.33%)	12 (24.00%)	29 (22.66%)	0.492
	CA	15 (50.00%)	32 (64.00%)	70 (54.69%)	
	AA	8 (26.67%)	6 (12.00%)	29 (22.66%)	
rs3796123	TT	13 (43.33%)	21 (42.00%)	67 (52.34%)	0.523
	TA	16 (53.33%)	28 (56.00%)	55 (42.97%)	
	AA	1 (3.33%)	1 (2.00%)	6 (4.69%)	
rs10202709	CC	17 (56.67%)	31 (62.00%)	83 (64.84%)	0.751
	CT	12 (40.00%)	17 (34.00%)	37 (28.91%)	
	TT	1 (3.33%)	2 (4.00%)	8 (6.25%)	

*p*—categorical Pearson’s chi-squared test.

**Table 5:** Haplotype frequencies across the five *FN1* SNVs.

Haplotypes	Frequency (Overall)	Frequency (Case/Ctrl)	Case/Ctrl *p*-Value	OC/Ctrl *p*-Value	EC/Ctrl *p*-Value	CC/Ctrl *p*-Value
GGATC	0.310	0.302, 0.318	0.6284	0.7021	0.5315	0.8294
GGCTT	0.125	0.100, 0.150	0.0299	0.0243	0.3660	0.3068
AGCAC	0.119	0.105, 0.133	0.2153	0.0433	0.3554	0.4060
GGCAC	0.084	0.096, 0.071	0.1998	0.2216	0.6622	0.0922
GGCTC	0.075	0.048, 0.103	0.0024	0.0067	0.0536	0.2301
GACTC	0.062	0.069, 0.056	0.4585	0.3128	0.4896	0.8076
AGATC	0.043	0.063, 0.022	0.0036	0.0071	0.0028	0.0269
GGATT	0.035	0.034, 0.036	0.8571	0.8457	0.3686	0.5833
AGCTT	0.026	0.032, 0.019	0.2312	0.0533	0.3124	0.9583
GAATC	0.026	0.034, 0.017	0.1080	0.2212	0.0959	0.3776
AGCTC	0.024	0.028, 0.020	0.4292	0.1775	0.7059	0.7415
AGAAC	0.019	0.020, 0.018	0.8110	0.7315	0.3594	0.3082
GGCAT	0.014	0.015, 0.014	0.8846	0.7353	0.8047	0.8499

*FN1* block containing rs35343655, rs1968510, rs6725958, rs3796123, and rs10202709.

**Table 6:** Information of selected SNVs in *FN1* gene.

rs Number	Position (GRCh38.p14)	Allele	Clinical Significance *	RegulomeDB Rank (Score)	1000Genomes EUR
rs35343655	chr2:215386974	G>A	Benign	1f (0.345)	G = 0.7485 A = 0.252
rs1968510	chr2:215393528	A>G	Benign	5 (0.590)	A = 0.101 G = 0.8989
rs6725958	chr2:215397898	A>C	Benign	4 (0.609)	A = 0.423 C = 0.578
rs3796123	chr2:215419235	T>A	Benign	1f (0.554)	T = 0.7366 A = 0.263
rs10202709	chr2:215433507	C>T	Benign	1d (0.373)	C = 0.7565 T = 0.244

* Clinical significance reported in ClinVar database. RegulomeDB variant ranks description: 1d—eQTL/caQTL + TF binding + any motif + chromatin accessibility peak; 1f—eQTL/caQTL + TF binding/chromatin accessibility peak; 4—TF binding + chromatin accessibility peak; 5—TF binding or chromatin accessibility peak.

**Table 7:** Primers and restriction enzymes used for RFLP analysis.

SNV	Primers	Restriction Enzyme	RFLP Products [bp]
rs35343655	5′-ACTgAAgTgCTCgggATgAT-3′ 5′-CAggAACgAAATgTTggATg-3′	MspI	A 236; C 139, 97
rs1968510	5′-gTTTgTTgTgTCAgTgTAgTA-3′ 5′-TgCATTAgCgTTATggCCATg-3′	TaqI	G 594, 190; A 784
rs6725958	5′-CTCAggACTTggATggTgTAgA-3′ 5′-TCATTTCCCAATAAAAgTACACTg-3′	HaeIII	A 256; C 171, 85
rs3796123	5′-ACCAATgCCAggATTCAgAg-3′ 5′-CCCAACTTAggCATgAgAgC-3′	AluI	A 152, 82; T 234
rs10202709	5′-CAgTCCCAgATCATggAgTCT-3′ 5′-gTACCATgTTACTTgTggAATAgAg-3′	HindIII	C 134, 68; T 206

## Data Availability

The original contributions presented in this study are included in the article. Further inquiries can be directed to the corresponding author.

## Abbreviations

The following abbreviations are used in this manuscript:

ASEs	alternative splicing events
BCG	Bacillus Calmette–Guérin
BMI	the body mass index
CC	cervical cancer
IIICS	the type III connecting segment ()
DNA	deoxyribonucleic acid
EC	endometrial cancer
ECM	the extracellular matrix
EMT	epithelial–mesenchymal transformation
EDA	the extra domains A
EDB	the extra domains B
EDTA	ethylenediaminetetraacetic acid
ELISA	enzyme-linked immunosorbent assay
FIGO	Federation of Gynecology and Obstetrics
FN	fibronectin
FN1	fibronectin 1 gene
GBM	glioblastoma multiforme
GWAS	genome-wide association studies
LD	linkage disequilibrium
cFN	cellular form in plasma
pFN	dimeric form in plasma
MAF	minor allele frequency
OC	ovarian cancer
OVCAR3	ovarian adenocarcinoma cell line
PCR-RFLP	the polymerase chain reaction-restriction fragment length polymorphism
PTC	premature termination codon
RFLP	restriction fragment length polymorphism
dbSNP	Database of Short Genetic Variations
SNVs	single-nucleotide variants
SPARC	secreted protein acidic and rich in cysteine
TME	the tumor microenvironment
TCGA	The Cancer Genome Atlas Program
